# Supplementary material for: An NKX-COUP-TFII morphogenetic code directs mucosal endothelial addressin expression
Source: Nat Commun. 2022 Dec 2;13:7448. doi: 10.1038/s41467-022-34991-2 (PMC9718832; doi:10.1038/s41467-022-34991-2)
Supplement: Supplementary file 1 — Supplementary Information [file 41467_2022_34991_MOESM1_ESM.pdf]

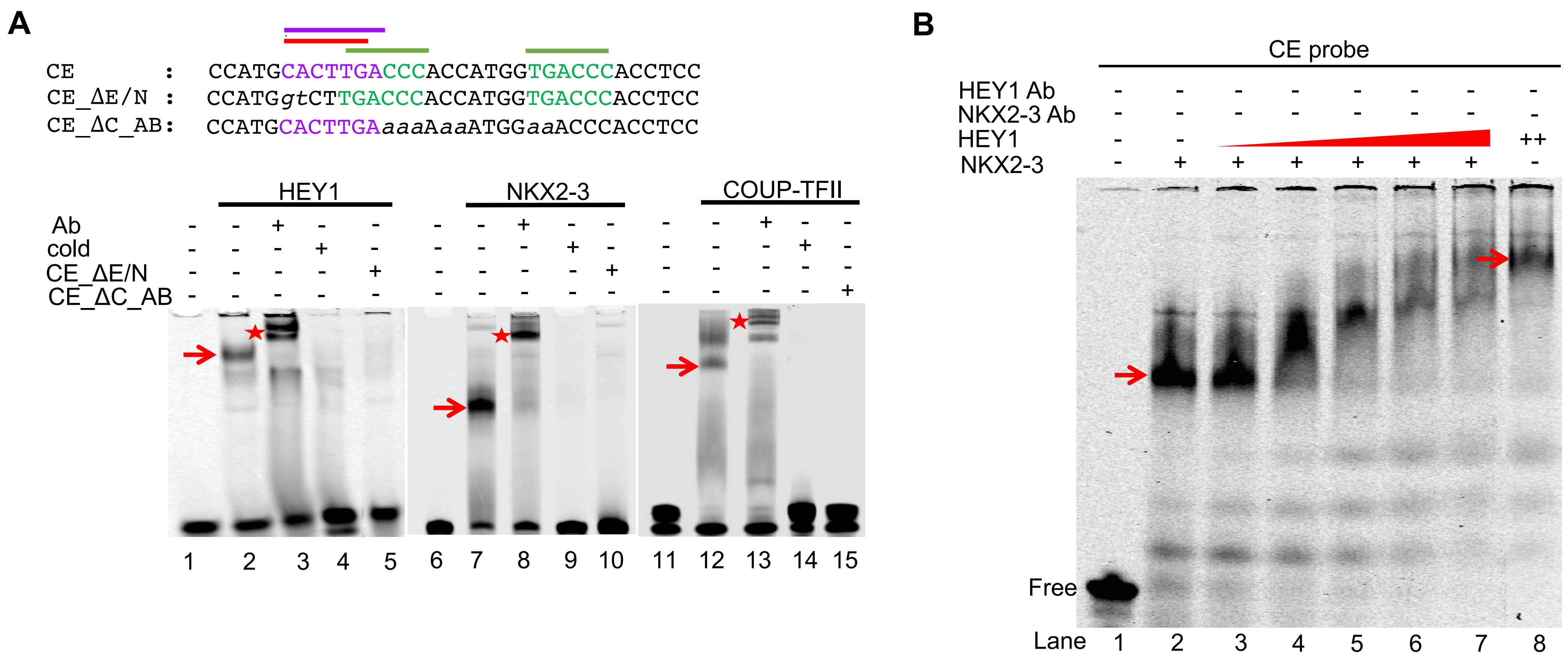

Figure S1. The *Madcam1* CE binds NKX2-3, COUP-TFII and HEY1.
(A) Sequence for the wildtype probe (CE) and mutant probes used for EMSA. CE_ΔE/N harbors mutations in the HEY1 and NKX2-3 binding site; CE_ΔC_AB harbors mutations in the COUP-TFII "A" and "B" sites. Red, purple and green bars denote the HEY1, NKX2-3 and COUP-TFII binding sites, respectively. EMSA showing migration of recombinant HEY1, NKX2-3 and COUP-TFII-GST bound to the CE probe (red arrows), supershifted by antibodies specific to the corresponding transcription factor (red stars). Cold competitor probe outcompeted the CE probe (lanes 4, 9, 14. Mutated probes lacking the HEY1 and NKX2-3 sites (CE_ΔE/N, lanes 5 and 10) or the COUP-TFII sites (CE_ΔC_AB, lane 15) failed to bind the respective TFs.
(B) EMSA showing migration of recombinant HEY1 and NKX2-3 bound to the CE probe (arrows). Progressive shift in migration indicates displacement of NKX2-3 by co-incubation with increasing concentrations of HEY1.

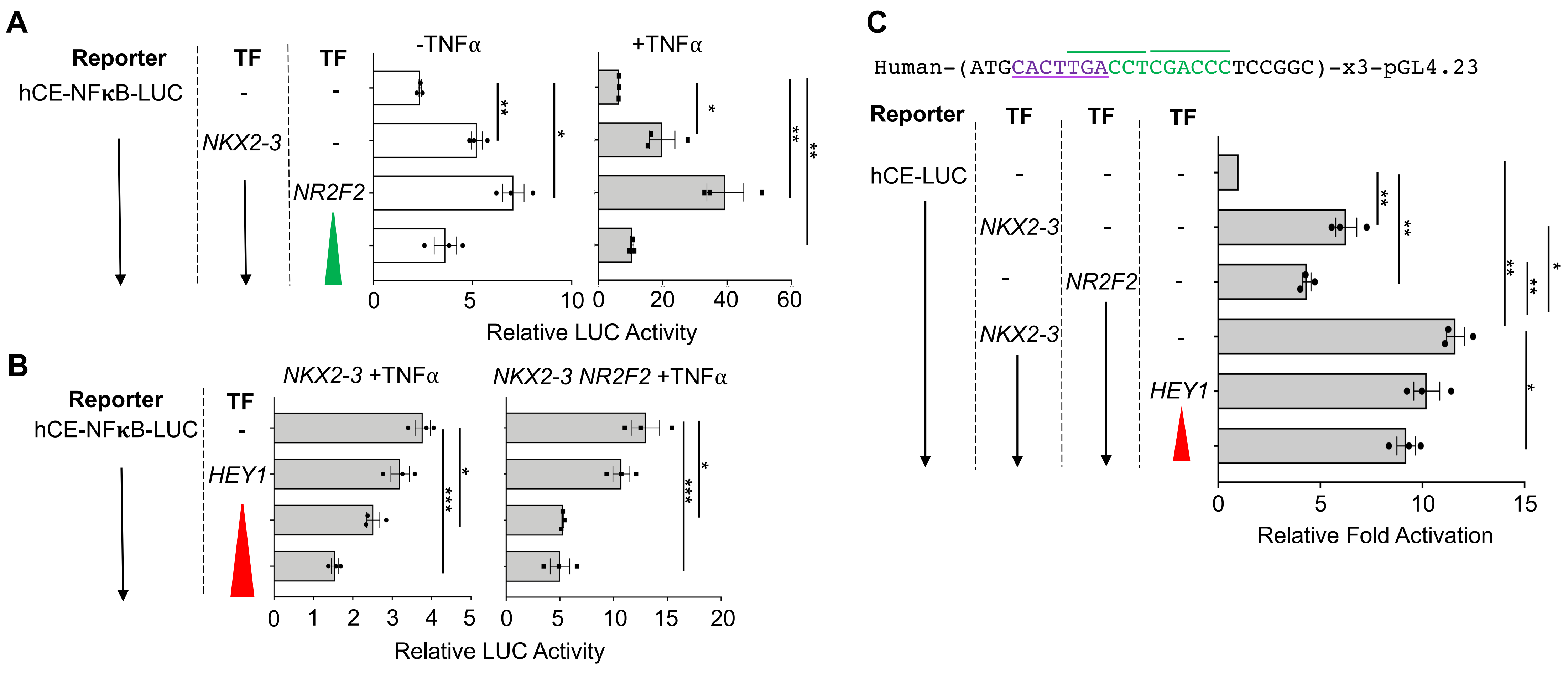

Figure S2. Functional properties of *MADCAM1* CE in human.
(A) Activity of luciferase reporter driven by CE-containing human *MADCAM1* promoter is enhanced by *NKX2-3* cooperatively with *NR2F2* in 293T cells.
(B) HEY1 dose-dependently suppresses activation of the *MADCAM1* reporter, when co-transfected with *NKX2-3* (left) or with *NKX2-3* and *NR2F2* (right) in 293T cells.
(C) Activity of luciferase reporter driven by promoter with three copies of the human *MADCAM1* CE sequence fused in tandem, when co-expressed with *NKX2-3*, *NR2F2* and *HEY1* in 293T cells.
All data are mean ± SEM of three independent experiments unless stated otherwise. ***: p-value <0.005, **: p-value<0.01, *: p-value<0.05. Two tailed t-test, paired.

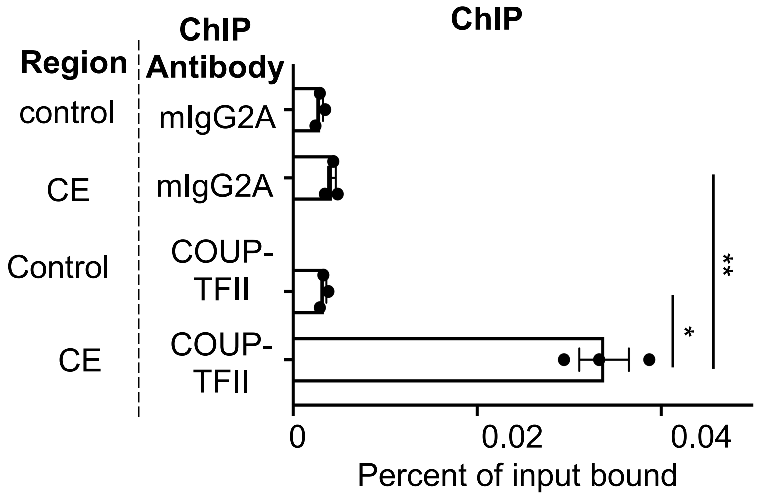

Figure S3. COUP-TFII binds to the endogenous *Madcam1* NCCE in DN-MAML bEnd.3 cells.
Anti-COUP-TFII chromatin immunoprecipitation from nuclei of DN-MAML bEnd.3 cells enriches for the CE. Real-time PCR using primers for the CE was performed on the input and immunoprecipitated DNA fractions and results were shown as percent of input CE DNA. Precipitation of an irrelevant genomic control region without a COUP-TFII binding site was used as a negative control. Results are representative of three independent experiments.

All data are mean ± SEM of three independent experiments unless stated otherwise. **: p-value<0.01, *: p-value<0.05. Two tailed t-test, paired.

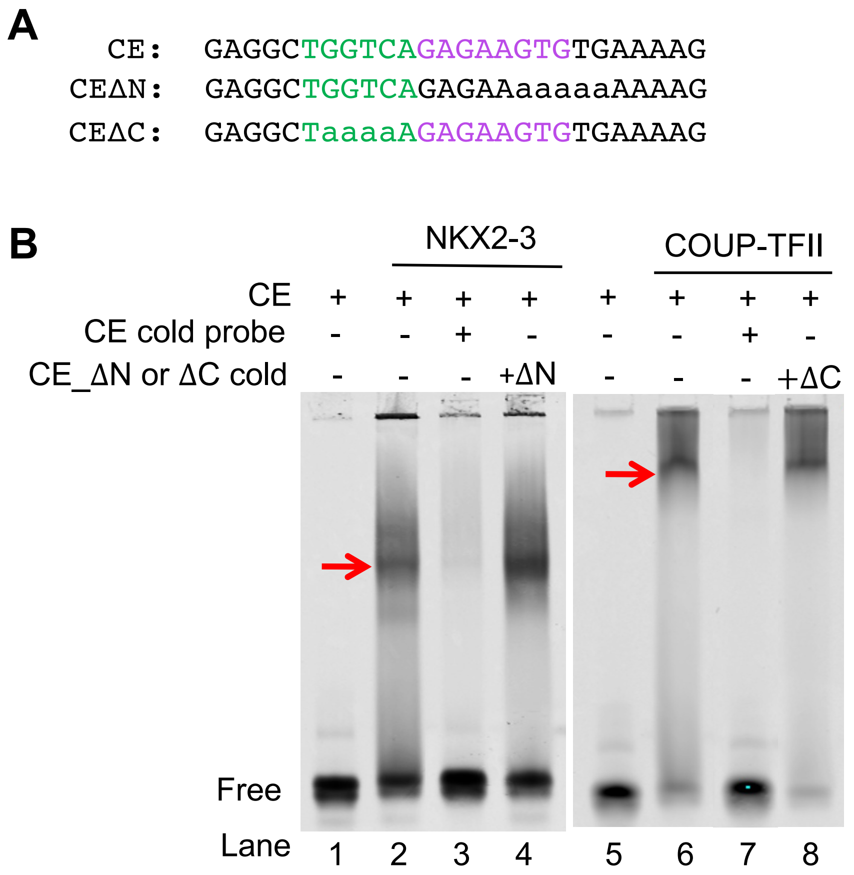

Figure S4. NKX2-3 and COUP-TFII bind the conserved *St6gal1* intronic NCCE

(A) Sequence of wildtype and mutant CE probes for EMSA. CE_ΔN harbors mutations in the NKX2-3 binding site; CE_ΔC harbors mutations in the COUP-TFII binding site.

(B) EMSA illustrating NKX2-3-HIS and COUP-TFII-GST recombinant proteins binding to the St6gal1 WT probe (red arrows). Cold WT probe outcompeted the CE probe, while mutated probes (CE_ΔN or CE_ΔC) failed to compete against the WT CE probe.

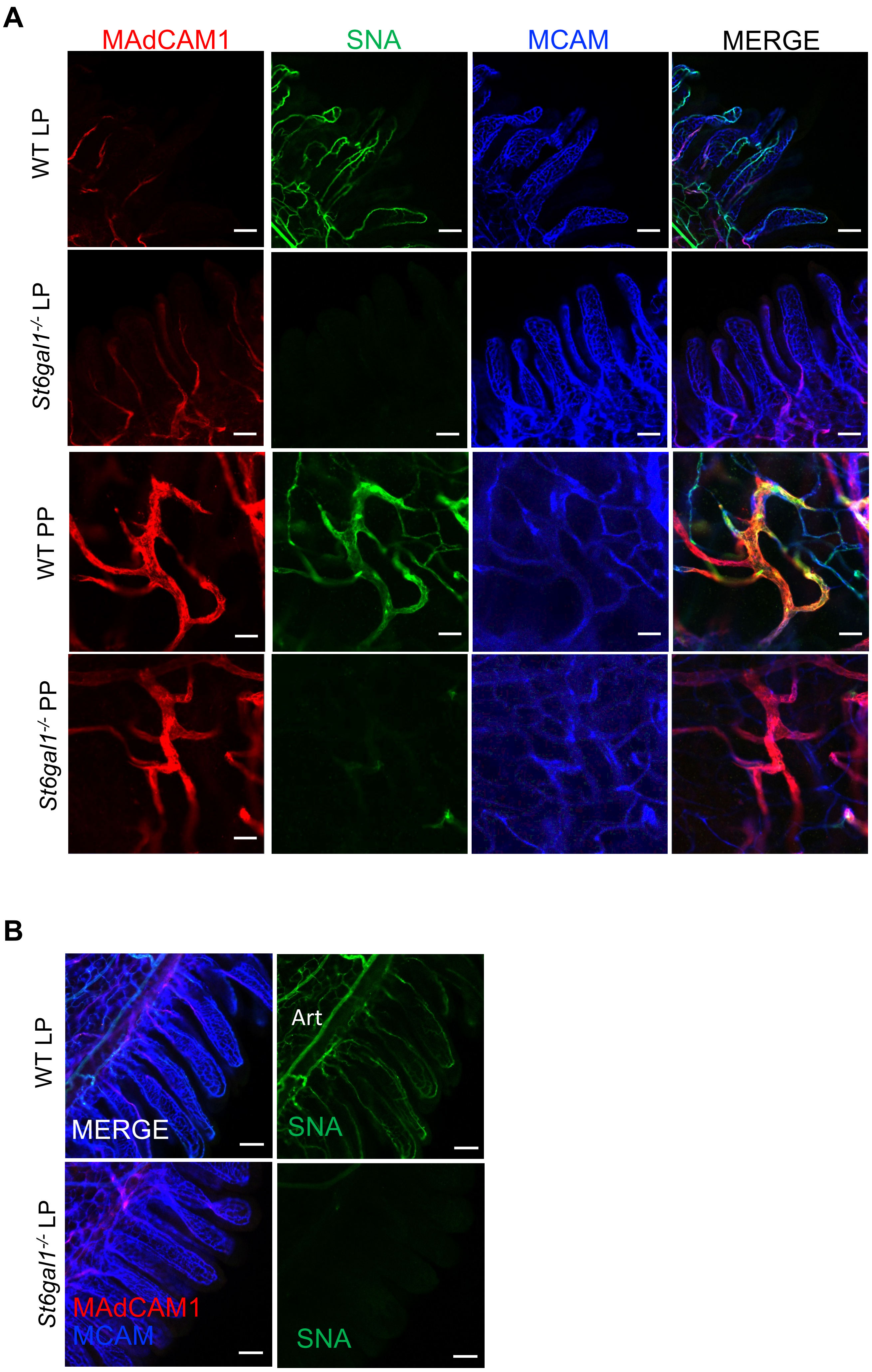

Figure S5. *St6gal*-dependent SNA binding to vascular endothelium in PP and LP.
(A) Binding of the alpha-2,6-sialic acid-specific lectin SNA to the LP and PP HEV in WT vs *St6gal1*$^{-/-}$ mice. Mice were injected with pan-EC anti-MCAM (CD146, blue) and AF450-labeled anti-addressin antibodies MECA89 and MECA367 to label HEV (red), followed by vascular prefusion with AF488-labeled SNA lectin (green). Scale bars: 100μm, 10x (top two rows), 50μm, 20x (bottom two rows); whole mount imaging.
(B) SNA stains the artery (Art) in WT but not *St6gal1*$^{-/-}$ mice. Whole mount, 10x, scale bars: 100μm.

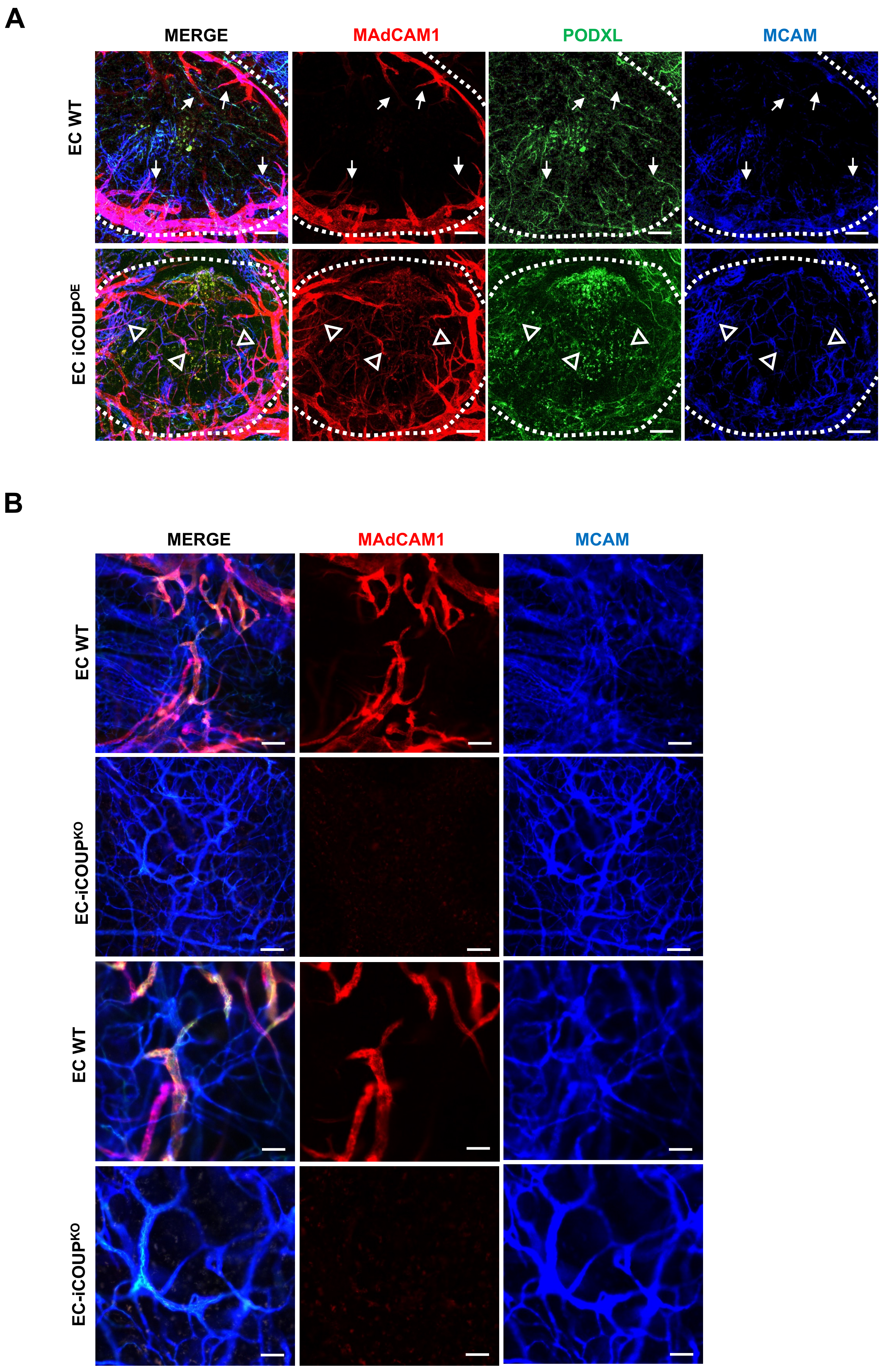

Figure S6. Abnormalities of MAdCAM1 expression in PP of in EC-iCOUP^{OE} and EC-iCOUP^{KO} mice.
(A) Confocal image showing MAdCAM1 extension from HEV into capillaries within the PP of EC-iCOUP^{OE} (arrowheads) but not control WT mice (arrows). Scale bars: 100μm, 20x, whole mount, 14 dpi.
(B) Confocal image showing distinct patterns of loss of MAdCAM1 in PP HEV in EC-iCOUP^{KO} vs control WT mice. Scale bars: 100μm, 10x (top two rows), 50μm, 20x (bottom two rows, same section as top two rows); whole mount imaging.

A

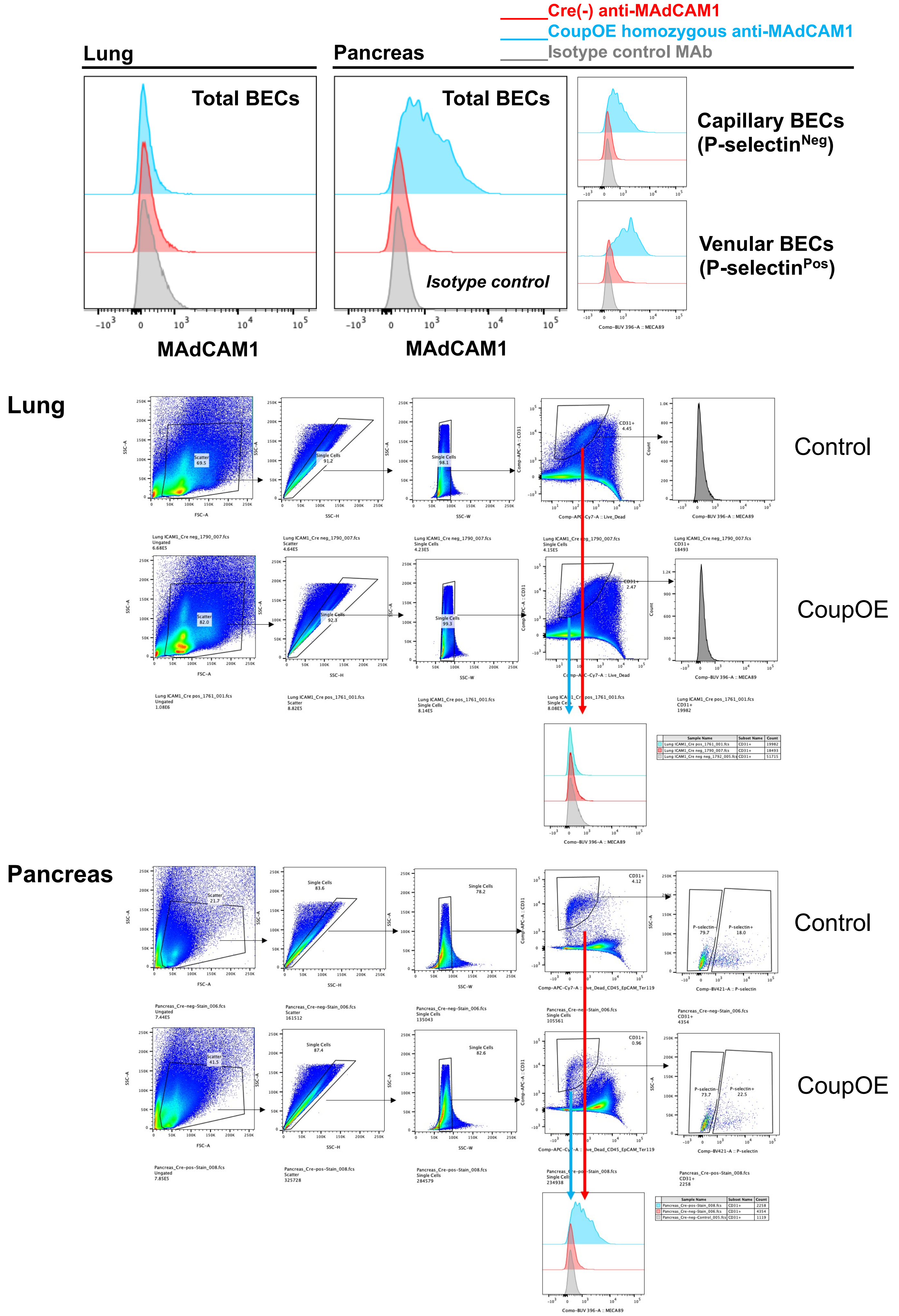

B

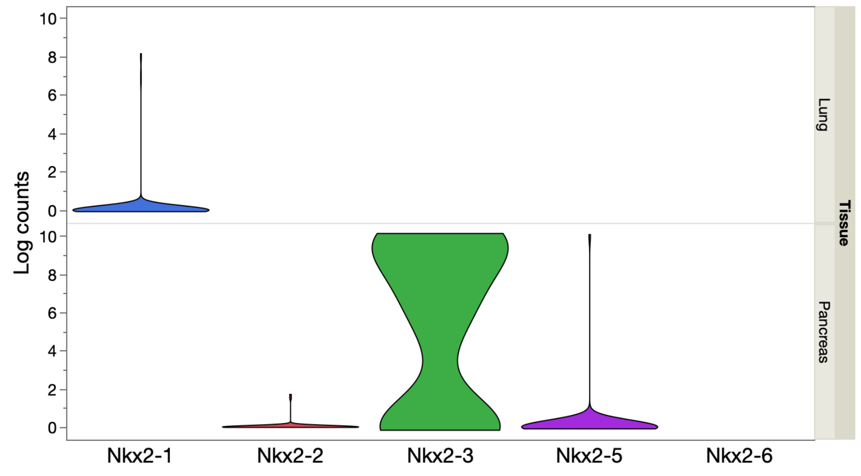

Figure S7. Ectopic expression of MAdCAM1 in the pancreas of EC-iCOUP^{OE} mice.

(A) Histograms showing absence of MAdCAM1 in lung BEC and ectopic expression of MAdCAM1 in pancreatic BEC in EC-iCOUP^{OE} mice and gating strategy for FACS.

(B) Violin plots of single-cell expression of *Nkx2* family genes in lung and pancreatic BEC (data from Tabula Muris Senis).

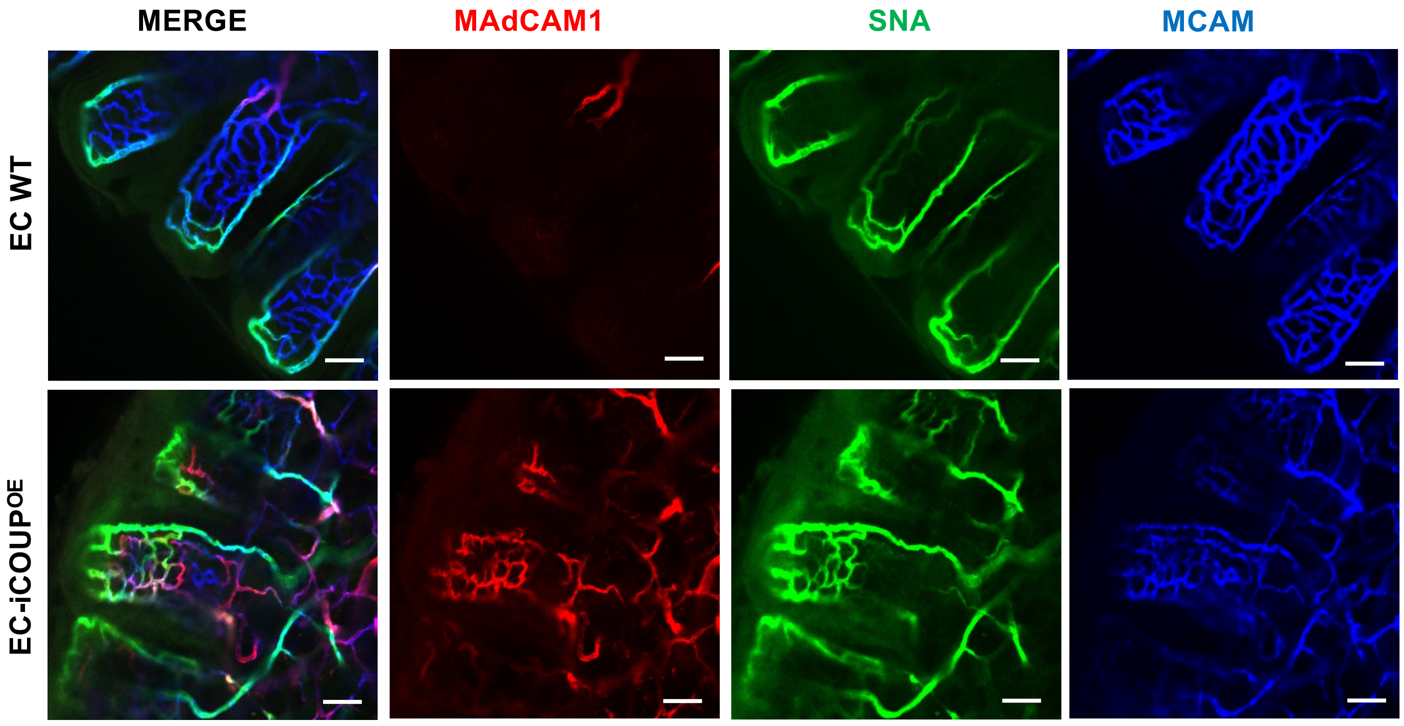

Figure S8. Ectopic co-expression of MAdCAM1 and SNA-binding glycotopes in the villus capillary network in EC-iCOUP^{OE} mice.
Confocal image showing distinct patterns of MAdCAM1 staining and SNA binding by lamina propria capillaries in EC-iCOUP^{OE} vs control WT mice. Scale bars: 50μm, 20x, whole mount, 19 dpi.

NKX:COUP-TFII motif in endocardial and atrial genes that control cell specification (*Etv2*, *Tbx5*)

Etv2

```
                                     NKX2-5          COUP-TFII
hEtv2Pro    chr19:35,642,021 CTGCAACTTGAGCCTGGCTGCGACCCCTG
cEtv2Pro    chr19:33,592,416 CTGCAACTTGAGCCTGGCTGCGACCCCTG
bEtv2Pro    chr19:35,062,091 CTGCAACTTGAGCCTGGCTGCGACCCCTG
raEtv2Pro   chr1: 89,093,789 CTGCAACTTGACCCAGGCTGCGACCCCCA
mEtv2Pro    chr7: 30,636,189 CTGCAACTTGACCCAGGCTGCGACCCCCA
                             *********** **:************ .
```

Tbx5

```
                                                 COUP-TFII     NKX2-5
hTbx5IVS   chr12:114,841,156  GGCCTGAATCGGCCGCTGACCTGGCCCTTATTAAGA
cTbx5IVS   chr12:112,068,880  GGCCTGAATCGGCCGCTGACCTGGCCCTTATTAAGA
rTbx5IVS   chr11:114,126,855  GGCCTGAAGCGGCCGCTGACCTGGCCCTTATTAAGA
raTbx5IVS  chr12:42,100,282   GGCCTGAAGCGGCCGCTGACCCGACCCTTAATAAG
mTBX5IVS   chr5:119,837,348   GGCCTGAAGCGGCCGCTGACCCGACCCTTAATAAGG
                              ******** ************ *.******:****.
```

NKX:COUP-TFII motif in interneuronal gene that controls cell migration (*Nrp2*)

Nrp2

```
                        NKX2-1         COUP-TFII
mNrp2IVS    GAATGTTGCCAAATTATGTGACCTTTGGCAGACCAGCTGTGC
raNrp2IVS   GAATGTTGCCAAATTATGTGACCTTTGGCAGACCAGCTGTGC
hNrp2IVS    GAACATTGCCAAATTATGTGACCTTTGGCAGACCAGCTGTGC
cNrp2IVS    GAACATTGCCAAATTATGTGACCTTTGGCAGACCAGCTGTGC
rNrp2IVS    GAACGTTGCCAAATTATGTGACCTTTGGCAGACCAGCTGTGC
            *** .*************************************
```

Figure S9. Conserved NCCE in select genes controlled by NKX and COUP-TFII in cardiac or neuronal development.
Alignment of candidate NCCE sequences from the promoter region of *Etv2* or the intronic regions of *Tbx5* and *Nrp2* across species. h, human; c, chimpanzee; b, baboon; r, rhesus monkey; ra, rat; m, mouse. Pro, promoter; IVS, intron. COUP-TFII and NKX family HD binding sites are highlighted in green and purple, respectively.

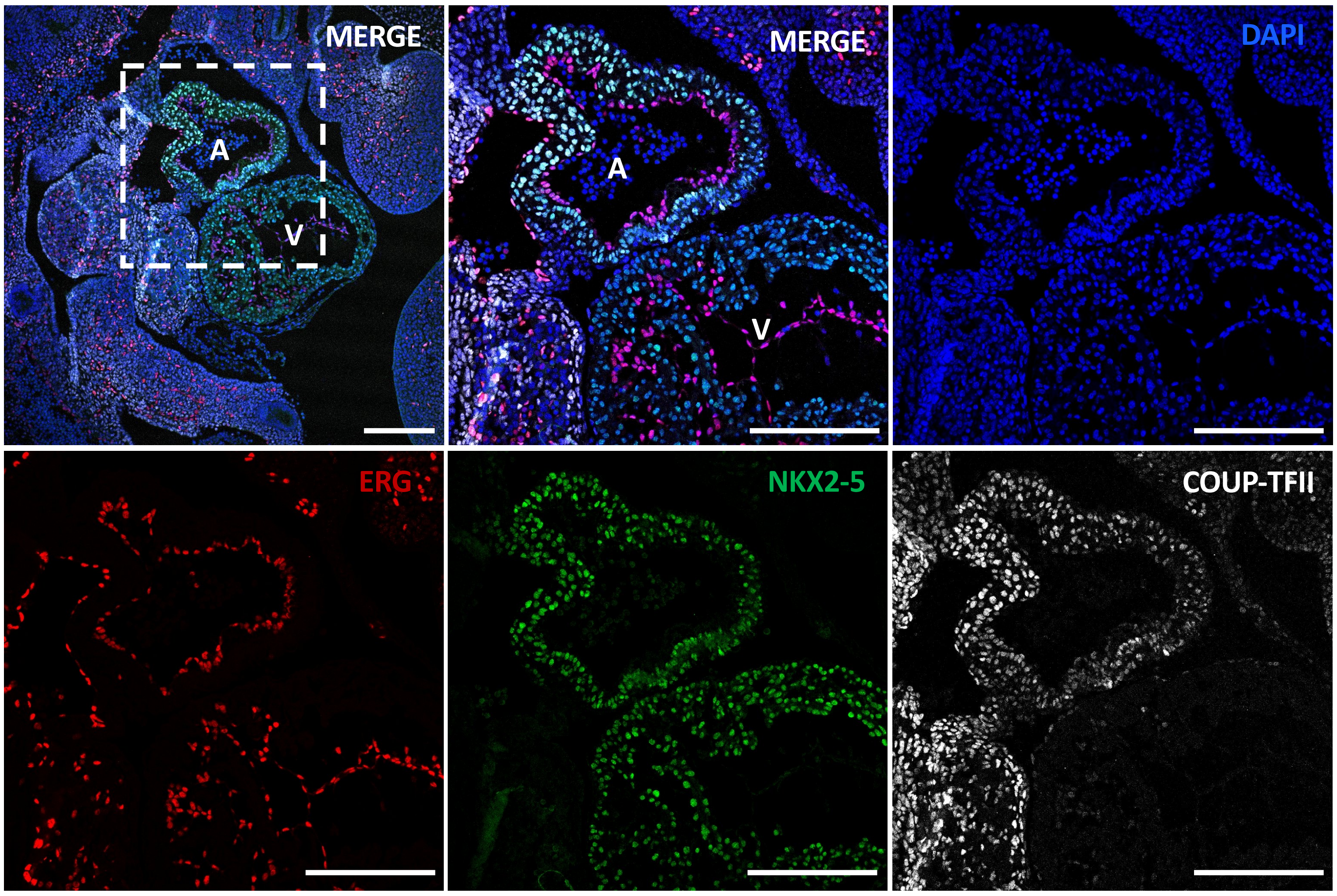

Figure S10. NKX2-5 and COUP-TFII co-localize in the developing mouse atrium.
Representative image of mouse heart (e10) stained for DAPI (blue), ERG (red), NKX2-5 (green) and COUP-TFII (white). Scale bars: 200 μm. A, atrium; V, ventricle.

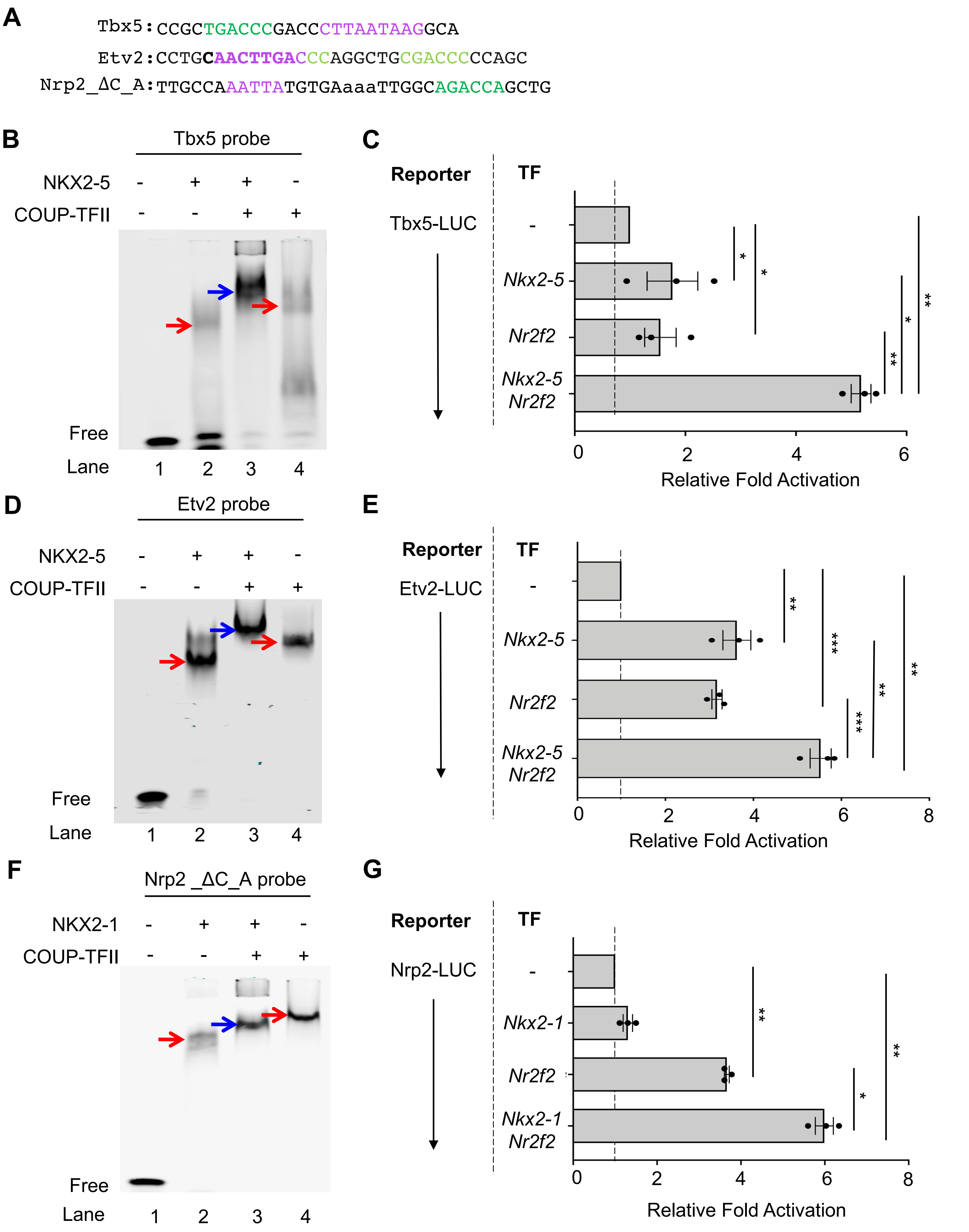

Figure S11. Conserved NCCE in *Tbx5*, *Etv2* and *Nrp2* promoters support combinatorial control of transcription by NKX2-5 or NKX2-1 and COUP-TFII.
(A) Sequence of probes used for EMSA.
(B, D, F) Binding of recombinant COUP-TFII and NKX2-5 or NKX2-1 to the *Tbx5* (B), *Etv2* (D) and *Nrp2* (F) NCCE probes. Heterodimeric complexes are indicated by blue arrows.
(C, E, G) Activity of luciferase reporters driven by NCCE-containing *Tbx5* (C), *Etv2* (E) or *Nrp2* (G) promoter, with or without *Nr2f2*, *Nkx2-5* or *Nkx2-1*.
All data are mean ± SEM from three independent experiments, each with 3 technical replicates. *: p-value<0.05, **: p-value <0.01, ***: p-value <0.005; two tailed t-tests, paired.

Supplementary Table S1. Primers and probes

Name	Sequence (5'-->3')	Application
MadR1_CE-F	CCATGCACTTGACCCACCATGGTGACCCACCTCC	EMSA
MadR1_CE-R	GGAGGTGGGTCACCATGGTGGGTCAAGTGCATGG	EMSA
MadR1_CE_ΔE/N-F	CCATGgtCTTGACCCACCATGGTGACCCACCTCC	EMSA
MadR1_CE_ΔE/N-R	GGAGGTGGGTCACCATGGTGGGTCAAGacCATGG	EMSA
MadR1_CE_ΔC_B-F	CCATGCACTTGACCCACCATGGgacGGCACCTCC	EMSA
MadR1_CE_ΔC_B-R	GGAGGTGCCgtcCCATGGTGGGTCAAGTGCATGG	EMSA
MadR1_CE_ΔC_A-F	CCATGCACTTGAaaaAaaATGGTGACCCACCTCC	EMSA
MadR1_CE_ΔC_A-R	GGAGGTGGGTCACCATttTtttTCAAGTGCATGG	EMSA
MadR1_CE_ΔC_AB-F	CCATGCACTTGAaaaAaaATGGaaAaaCACCTCC	EMSA
MadR1_CE_ΔC_AB-R	GGAGGTGttTttCCATttTtttTCAAGTGCATGG	EMSA
MadR1_CE_ΔN_ΔC_A-F	CCATGgtCTTGAaaaAaaATGGTGACCCACCTCC	EMSA
MadR1_CE_ΔN_ΔC_A-R	GGAGGTGGGTCACCATttTtttTCAAGacCATGG	EMSA
mETV2-F	CCTGCAACTTGACCCAGGCTGCGACCCCCAGC	EMSA
mETV2-R	GCTGGGGGTCGCAGCCTGGGTCAAGTTGCAGG	EMSA
mSt6gal1 CE-F	GAGGCTGGTCAGAGAAGTGTGAAAAG	EMSA
mSt6gal1 CE-R	CTTTTCACACTTCTCTGACCAGCCTC	EMSA
mSt6gal1 CE_ΔN-F	GAGGCTGGTCAGAGAAaaaaaAAAAG	EMSA
mSt6gal1 CE_ΔN-R	CTTTTtttttTTCTCTGACCAGCCTC	EMSA
mSt6gal1 CE_ΔC-F	GAGGCTaaaaAGAGAAGTGTGAAAAG	EMSA
mSt6gal1 CE_ΔC-R	CTTTTCACACTTCTCTttttAGCCTC	EMSA
NRP2-F	TTGCCAAATTATGTGACCTTTGGCAGACCAGCTG	EMSA
NRP2-R	CAGCTGGTCTGCCAAAGGTCACATAATTTGGCAA	EMSA
Nrp2_ΔC-F	TTGCCAAATTATGTGAaaaTTGGCAaaaaAGCTG	EMSA
Nrp2_ΔC-R	CAGCTttttTGCCAAtttTCACATAATTTGGCAA	EMSA
Nrp2_ΔN-F	TTGCgtAAggAgGTGACCTTTGGCAGACCAGCTG	EMSA
Nrp2_ΔN-R	CAGCTGGTCTGCCAAAGGTCACcTccTTacGCAA	EMSA
Nrp2_ΔC_A-F	TTGCCAAATTATGTGAaaaTTGGCAGACCAGCTG	EMSA
Nrp2_ΔC_A-R	CAGCTGGTCTGCCAAtttTCACATAATTTGGCAA	EMSA
Tbx5-F	CCGCTGACCCGACCCTTAATAAGGCA	EMSA
Tbx5-R	TGCCTTATTAAGGGTCGGGTCAGCGG	EMSA
Tbx5_ΔC-F	CCGCTGAaaaaACCCTTAATAAGGCA	EMSA
Tbx5_ΔC-R	TGCCTTATTAAGGGTttttTCAGCGG	EMSA
Tbx5_1m2-F	CCGCTGACCCGACCCTggggAAGGCA	EMSA
Tbx5_1m2-R	TGCCTTccccAGGGTCGGGTCAGCGG	EMSA
NRP2-F	TTGCCAAATTATGTGACCTTTGGCAGACCAGCTG	EMSA
NRP2-R	CAGCTGGTCTGCCAAAGGTCACATAATTTGGCAA	EMSA
Hey1_pet21-F	CGGGATCCatgaagagagctcacccagac	for pet21A cloning; has BamHI site
Hey1_pet21-R	TCGGAATTCCGgaaagctccgatctctgtc	for pet21A cloning; has EcoRI site+2nt to keep in frame
nkx_pet21-F	CGGAATTCatgatgttaccaag cccggtc	for pet21A cloning; has EcoRI site
nkx_pet21rev-R	TCAAGCTTCccaagccctgatgccctgcaaag	for pet21A cloning; has HindIII site +1nt to keep in frame
nr2f2/coup_pet21-F	CGGGATCCatggca atggtagtca gcacg	for pet21A cloning; has BamHI site
nr2f2/coup_pet21-R	TCGGAATTCCGttgaattgccatatatggc	for pet21A cloning; has EcoRI site+2nt to keep in frame
nr2f2_pgex2TK-R	CCGGAATTCCGGttgaattgccatatatggc	for pGEX2TK cloning has EcoRI site; use nr2f2_pet21A-F for forward primer which has BamhI site
nkx2.3Δ2_24_pet21A-F	CGGAATTCatgcacttccacggagctcacttgc	for pet21A cloning; has EcoRI site; has aa2-24 missing added atg; use nkxpet21rev-R primer
nkx2-5_pet21A-F	CGGGATCCatgttccccag ccctgcgctc acac	for pet21A cloning; has BamHI site
nkx2-5_pet21A-R	TCGGAATTCCGccaggctcggatgccgtgcagcgtg	for pet21A cloning; has EcoRI site+2nt to keep in frame
mNFKBCE_LUC pGL4.10-F	aaaggtaccGCACCATCCAGAACCAGGCCAG	has KpnI site; NFKBCE_LUC for LUC reporter
mNFKBCE_LUC pGL4.10-F	aaaaagcttCTCTGTCTTGTGACCGCCAGGGGAAG	has HindIII site; NFKBCE_LUC for LUC reporter
mCE_LUC pGL4.10-F	CTCGGTACCgtacatagaaccggggcc	has KpnI site; CE_LUC for LUC reporter
mCE_LUC pGL4.10-R	aaaaagcttCTCTGTCTTGTGACCGCCAGGGGAAG	has HindIII site; CE_LUC for LUC reporter
mMAD_SDMm1-F	gactggcagatttccatgGTcttgacccaccatggtga	mutated Nkx binding site; LUC reporter
mMAD_SDMm1-R	tcaccatggtgggtcaagACcatggaaatctgccagtc	mutated Nkx binding site; LUC reporter
mMAD_SDMm5-F	ccatgcacttgaAAAaAAatggAAaAAcacctccagcttttag	mutated COUP-TFII binding site; LUC reporter
mMAD_SDMm5-R	ctaaaagctggaggtgTTtTTccatTTtTTTtcaagtgcatgg	mutated COUP-TFII binding site; LUC reporter
MadChIP_R1-F	actcaggccccagaacaact	ChIP-qPCR
MadChIP_R1-R	tacgggaaggctgaagctaa	ChIP-qPCR
GenCtrl4-F	ggatgggcgcacgtacag	ChIP-qPCR; 3' of TPGS (genomic control)
GenCtrl4-R	acgaattccagcagcacaaa	ChIP-qPCR; 3' of TPGS (genomic control)
mMad_SDMPCR-F	GGCACAGCTCTAGTCAGCGAGGTG	Colony PCR for SDM of LUC reporter
mMad_SDMPCR-R	GAACACAGTCATTACAGATGGGGA	Colony PCR for SDM of LUC reporter
MadqRT-PCR-Hel-F	CTGAGCCCTACATCCTGACCT	Real-Time RT-PCR Madcam1
MadqRT-PCR-Hel-R	GCTTCACAGAGTAGCTCCCAG	Real-Time RT-PCR Madcam1
hCE_NFκB LUC pGL4.10	GGACCCGCCGTCGGGGTGGGGGCGG	Start sequence for custom Cyagen vector
**Commercial primers**		
Sino Biological	MP200715	ST6GAL1 qPCR Primer Pairs, Mouse
Qiagen	PPM02945B-200	b actin qPCR Primer Pairs, Mouse

Supplementary Table S2. List of Antibodies

Name	Company	Cat #	Application	Dilution
Nkx2-3 (O-21)	Santa Cruz Biotechnology	sc-133826	WB	1:100
Nkx2-3 (S-16)	Santa Cruz Biotechnology	sc-83438x	EMSA	1:10
Nkx2-3	Atlas Antibodies	HPA047561	IHC, PLA, IF	1:100 (IHC & IF); 1:75 (PLA)
COUP-TFII	Perseus Proteomics	PP-H7147-00	IHC, PLA, ChIP, WB, IF	1:100 for everything except EMSA 1:10
MAdCAM1 (MECA367)	in house	N/A	IF, ELISA	1:100; 25ug (IF)
MAdCAM1 (MECA89)	in house	N/A	FACS, IF	1:50 (FACS); 25ug (IF)
PLVAP (MECA32)	in house	N/A	ELISA	1:100
MAdCAM1	R&D Systems	AF-993	IHC, WB	1:100
Erg-488	Abcam	196374	PLA	1:100
Erg-647	Abcam	ab196149	IF	1:100
CD146	BD Biosciences	740095	IF	30uL injected
ly6c apc cy7	biolegend	128026	FACS	1:100
gp38 PeCy7 Ab	Biolegend	127412	FACS	1:100
cd11b PerCP cy5.5 ab	eBioscience	550993	FACS	1:100
Cd11a/cd18 PerCP Cy5.5 Ab	Biolegend	141008	FACS	1:100
CD45 PerCP Cy5.5 Ab	Biolegend	103132	FACS	1:100
Nkx2-1 Ab	Biopat Immunotechnologies	PA0100	EMSA	1:10
nkx2-5 Ab	Santa Cruz	sc-376565X	EMSA	1:10
PerCP/Cy5.5 anti-mouse TER-119/Erythroid Cells Antibody	Biolegend	116228	FACS	1:100
CD31 BV605	Biolegend	102427	FACS	1:100
SIRPa PerCP cy5.5 Ab	Biolegend	144009	FACS	1:100
M99 (Podoplanin) 633	In house		FACS	1:100
ttf-1 (NKX2-1 AB)	Santa Cruz	SC-53136	EMSA	1:10
percpCy5.5 anti-mouse CD326 (EPCAM) clone G8.8	Biolegend	118219	FACS	1:100
Sambucus Nigra Lectin (SNA, EBL), Fluorescein	Vector laboratories	FL-1301-2	IF	3ug/ml
SNA-Cy3	Vector laboratories	CL-1303	IF	1:100
Erg	Abcam	ab92513	IF	1:100
Nkx2-5	Santa Cruz	sc-8697	IF	1:100
M79-DL633	In house		IF	25ug
Prolong Gold antifade reagent	Invitrogen	P36934	IF	

Supplementary Table S3. List of plasmids

Name	Company	Cyagen Vector ID
mCE_NFκB LUC pGL4.10	in house	
mNFκB-LUC pGL4.10	in house	
Nkx2-3	ABM	
Nr2f2	ABM	
Hey1	ABM	
NKX2-3 pET21A	in house	
HEY1-pET21A	in house	
COUP-TFII-pET21A	in house	
COUP-TFII-pGEX2TK	in house	
NKX2-1 pET21A	Cyagen	VB150629-10021
NKX2-5 pET21A	in house	
pLV[Exp]-EGFP:T2A:Puro-EF1A>{mNkx2-3[NM_008699.2]*(1-24aa)	Cyagen	VB170302-1081wyz
eGFP vector control for Nkx-TN OE; pLV[Exp]-Puro-CMV>EGFP	Cyagen	VB170302-1030htt
Nkx2-3 shRNA lines	Origene	
Nr2f2 shRNA lines	Origene	
Control shRNA line	Origene	
St6Gal1-LUC pGl4.23	Cyagen	VB190420-1028twv
St6Gal1ΔCoup-LUC pGL4.23	Cyagen	VB190420-1029bwr
CE_ΔN NFκB LUC pGL4.10	in house	
CE_ΔC_A LUC pGL4.10	Cyagen	VB190513-1157phx
CE_ΔC_B LUC pGL4.10	Cyagen	VB190513-1160uqw
mCE-LUC pGL4.23	Cyagen	VB190513-1166pry
hCE-LUC pGL4.23	Cyagen	VB190513-1167ruw
Nrp2-LUC pGL4.23	Cyagen	VB190420-1031gqm
Tbx5-LUC pGL4.23	Cyagen	VB190420-1033frx
hCE_NFκB LUC pGL4.10	Cyagen	VB190513-1159whr
pGL4.23-minP>luc2	Cyagen	VB190507-1247jqg
