# Supplementary material for: An NKX-COUP-TFII morphogenetic code directs mucosal endothelial addressin expression
Source: Nat Commun. 2022 Dec 2;13:7448. doi: 10.1038/s41467-022-34991-2 (PMC9718832; doi:10.1038/s41467-022-34991-2)
Supplement: Supplementary file 3 — Description of Additional Supplementary Files [file 41467_2022_34991_MOESM3_ESM.pdf]

Description of Additional Supplementary Files

File Name: Supplementary Data 1
Description: NCCE loci in the mouse and human genomes.
