# Supplementary material for: An NKX-COUP-TFII morphogenetic code directs mucosal endothelial addressin expression
Source: Nat Commun. 2022 Dec 2;13:7448. doi: 10.1038/s41467-022-34991-2 (PMC9718832; doi:10.1038/s41467-022-34991-2)
Supplement: Supplementary file 5 — Reporting Summary [file 41467_2022_34991_MOESM5_ESM.pdf]

nature portfolio

Corresponding author(s): Eugene C. Butcher

Last updated by author(s): Nov 7, 2022

Reporting Summary

Nature Portfolio wishes to improve the reproducibility of the work that we publish. This form provides structure for consistency and transparency in reporting. For further information on Nature Portfolio policies, see our Editorial Policies and the Editorial Policy Checklist.

Statistics

For all statistical analyses, confirm that the following items are present in the figure legend, table legend, main text, or Methods section.

n/a	Confirmed	
☐	☒	The exact sample size (*n*) for each experimental group/condition, given as a discrete number and unit of measurement
☐	☒	A statement on whether measurements were taken from distinct samples or whether the same sample was measured repeatedly
☐	☒	The statistical test(s) used AND whether they are one- or two-sided<br>*Only common tests should be described solely by name; describe more complex techniques in the Methods section.*
☐	☒	A description of all covariates tested
☐	☒	A description of any assumptions or corrections, such as tests of normality and adjustment for multiple comparisons
☐	☒	A full description of the statistical parameters including central tendency (e.g. means) or other basic estimates (e.g. regression coefficient) AND variation (e.g. standard deviation) or associated estimates of uncertainty (e.g. confidence intervals)
☐	☒	For null hypothesis testing, the test statistic (e.g. *F*, *t*, *r*) with confidence intervals, effect sizes, degrees of freedom and *P* value noted<br>*Give P values as exact values whenever suitable.*
☒	☐	For Bayesian analysis, information on the choice of priors and Markov chain Monte Carlo settings
☒	☐	For hierarchical and complex designs, identification of the appropriate level for tests and full reporting of outcomes
☒	☐	Estimates of effect sizes (e.g. Cohen's *d*, Pearson's *r*), indicating how they were calculated

Our web collection on statistics for biologists contains articles on many of the points above.

Software and code

Policy information about availability of computer code

Data collection	Luciferase assays were carried out using the Dual-Glo Luciferase assay system (Promega) and a Turner, TD-20/20 luminometer. All gels were imaged using the Odyssey Imaging System from Licor. The input and ChIP samples were subjected to real-time PCR on a Biorad thermocycler. Single cell gene expression was assayed using the 10x Chromium v3 platform using Chromium Single Cell 3' Library and Gel Bead Kit v2 (10X Genomics, PN-120237) according to 10X Genomics guidelines. Male and female cohorts were processed together and resolved post-sequencing. Libraries were sequenced on an Illumnia NextSeq 500 using 150 cycles high output V2 kit (Read 1–26, Read2-98 and Index 1–8 bases).
Data analysis	The Cell Ranger package (v3.0.2) was used to align high quality reads to the mm10 transcriptome (quality control reports available: https://stanford.io/37sXZV3). Quality control and data analysis was carried out as described. Briefly, normalized log expression values were calculated using the scran package. Imputed expression values were calculated using a customized implementation (https://github.com/kbrulois/magicBatch) of the MAGIC (Markov Affinity-based Graph Imputation of Cells) algorithm and optimized parameters (t = 2, k = 9, ka = 3). Supervised cell selection was used to remove cells with non-blood endothelial cell gene signatures: lymphatic endothelial cells (Prox1, Lyve1, Pdpn); Pericytes (Itga7, Pdgfrb); fibroblastic reticular cells (Pdpn, Ccl19, Pdgfra); lymphocytes (Ptprc, Cd52). The Arterial (Gkn3+), HEC, PCV and CRP clusters were defined based on canonical marker expression. Batch effects from technical replicates were removed using the MNN algorithm as implemented in the batchelor package's (v1.0.1) fastMNN function. Dimensionality reduction was performed using the UMAP algorithm. For UMAP embeddings, cell-cycle effects were removed by splitting the data into dividing and resting cells and using the fastMNN function to align the dividing cells with their resting counterparts. Violin plots were generated using ggplot2. Conserved genomic regions were defined as regulatory elements with log-odds score greater than 300 in UCSC Genome Browser's phastCons Placental Elements track, which aligns 60 vertebrate species and measures evolutionary conservation of genomic sequences. COUP-TFII and NKX motifs were searched within each conserved region in the mouse genome using HOMER (v4.11). The spacing between the two motifs within a NCCE was calculated as the distance between the centers of the motifs. Genome coordinates of the motifs were converted from mouse (mm10) to human (hg38) using

the liftOver tool, and only mouse coordinates whose syntenic human counterparts correspond to COUP-TFII and NKX binding motifs were defined as conserved NCCE. Custom scripts for identifying conserved NCCEs are available at https://github.com/mxiang1/NCCE.

For manuscripts utilizing custom algorithms or software that are central to the research but not yet described in published literature, software must be made available to editors and reviewers. We strongly encourage code deposition in a community repository (e.g. GitHub). See the Nature Portfolio guidelines for submitting code & software for further information.

Data

Policy information about availability of data

All manuscripts must include a data availability statement. This statement should provide the following information, where applicable:

- Accession codes, unique identifiers, or web links for publicly available datasets
- A description of any restrictions on data availability
- For clinical datasets or third party data, please ensure that the statement adheres to our policy

The authors declare that all data supporting the findings of this study are available within the article and its supplementary information files.
Genome databases used: mouse (mm10) and human (hg38).

Human research participants

Policy information about studies involving human research participants and Sex and Gender in Research.

Reporting on sex and gender	N/A
Population characteristics	N/A
Recruitment	N/A
Ethics oversight	N/A

Note that full information on the approval of the study protocol must also be provided in the manuscript.

Field-specific reporting

Please select the one below that is the best fit for your research. If you are not sure, read the appropriate sections before making your selection.

☒ Life sciences ☐ Behavioural & social sciences ☐ Ecological, evolutionary & environmental sciences

For a reference copy of the document with all sections, see nature.com/documents/nr-reporting-summary-flat.pdf

Life sciences study design

All studies must disclose on these points even when the disclosure is negative.

Sample size	No method was used to predetermine sample size. A minimum of 3 biological replicates were used for each experiment since it is necessary to achieve statistical significance.
Data exclusions	For scRNAseq, supervised cell selection was used to remove cells with non-blood endothelial cell gene signatures: lymphatic endothelial cells (Prox1, Lyve1, Pdpn); Pericytes (Itga7, Pdgfrb); fibroblastic reticular cells (Pdpn, Ccl19, Pdgfra); lymphocytes (Ptprc, Cd52). Low-quality cells were also excluded from analysis.
Replication	All attempts at replication were successful.
Randomization	Male and female mice of similar age were randomly allocated to each experimental group.
Blinding	Researchers were not blinded but were as unbiased as possible when acquiring and analyzing data.

Reporting for specific materials, systems and methods

We require information from authors about some types of materials, experimental systems and methods used in many studies. Here, indicate whether each material, system or method listed is relevant to your study. If you are not sure if a list item applies to your research, read the appropriate section before selecting a response.

Materials & experimental systems

n/a	Involved in the study
☐	☒ Antibodies
☐	☒ Eukaryotic cell lines
☒	☐ Palaeontology and archaeology
☐	☒ Animals and other organisms
☒	☐ Clinical data
☒	☐ Dual use research of concern

Methods

n/a	Involved in the study
☒	☐ ChIP-seq
☒	☐ Flow cytometry
☒	☐ MRI-based neuroimaging

Antibodies

Antibodies used

Nkx2-3 (O-21) Santa Cruz Biotechnology sc-133826 WB
Nkx2-3 (S-16) Santa Cruz Biotechnology sc-83438x EMSA
Nkx2-3 (H-54) Santa Cruz Biotechnology sc-292289x co-IP
Nkx2-3 Atlas Antibodies HPA047561 IHC, PLA, IF
COUP-TFII Perseus Proteomics PP-H7147-00 IHC, PLA, ChIP, WB, IF
MAdCAM1 (MECA367) in house N/A IF, ELISA
MAdCAM1 (MECA89) in house N/A FACS, IF
PLVAP (MECA32) in house N/A ELISA
MAdCAM1 R&D Systems AF-993 IHC, WB
Erg-488 Abcam 196374 PLA
Erg-647 Abcam ab196149 IF
CD146 BD Biosciences 740095 IF
ly6c apc cy7 biolegend 128026 FACS
gp38 PeCy7 Ab Biolegend 127412 FACS
cd11b PerCP cy5.5 ab eBioscience 45-0112-82 FACS
Cd11a/cd18 PerCP Cy5.5 Ab Biolegend 141008 FACS
CD45 PerCP Cy5.5 Ab Biolegend 103132 FACS
Nkx2-1 Ab Biopat Immunotechnologies PA0100 EMSA
nkx2-5 Ab Santa Cruz sc-376565X EMSA
PerCP/Cy5.5 anti-mouse TER-119/Erythroid Cells Antibody Biolegend 116228 FACS
CD31 BV605 Biolegend 102427 FACS
SIRPa PerCP cy5.5 Ab Biolegend 144009 FACS
ttf-1 (NKX2-1 AB) Santa Cruz SC-53136 EMSA
percpCy5.5 anti-mouse CD326 (EPCAM) clone G8.8 Biolegend 118219 FACS
Sambucus Nigra Lectin (SNA, EBL), Fluorescein Vector laboratories FL-1301-2 IF
SNA-Cy3 Vector laboratories CL-1303 IF
Erg Abcam ab92513 IF
Nkx2-5 Santa Cruz sc-8697 IF
Prolong Gold antifade reagent Invitrogen P36934 IF

Validation

Nkx2-3 (O-21) Santa Cruz Biotechnology sc-133826 WB Validated by company via WB; https://datasheets.scbt.com/sc-133826.pdf
Citations: Harvey, R.P. 1996. NK2 homeobox genes and heart development. Dev. Biol. 178: 203-216; Ray, M.K., Chen, C.Y., Schwartz, R.J. and DeMayo, F.J. 1996. Transcriptional regulation of a mouse Clara cell-specific protien (mCC10) gene by the Nkx transcription factor family members thyroid transciption factor 1 and cardiac muscle-specific homeobox protein (CSX). Mol. Cell. Biol. 16: 2056-2064.
Nkx2-3 (S-16) Santa Cruz Biotechnology sc-83438x EMSA Validated by company; no longer made, tested in house and by company via Western Blot
Nkx2-3 Atlas Antibodies HPA047561 IHC, PLA, IF Validated by company via IHC; https://www.atlasantibodies.com/products/antibodies/primary-antibodies/triple-a-polyclonals/nkx2-3-antibody-hpa047561/ https://www.proteinatlas.org/ENSG00000119919-NKX2-3/tissue/primary+data
COUP-TFII Perseus Proteomics PP-H7147-00 IHC, PLA, ChIP, WB, IF Validated by company via WB, IHC, IP; https://www.rndsystems.com/products/human-coup-tf-ii-nr2f2-antibody-h7147_pp-h7147-00 "Citations: BRN2 as a key gene drives the early primate telencephalon development
Authors: X Zhu, Y Guo, C Chu, D Liu, K Duan, Y Yin, C Si, Y Kang, J Yao, X Du, J Li, S Zhao, Z Ai, Q Zhu, W Ji, Y Niu, T Li
Science Advances, 2022;8(9):eabl7263.
Species: Primate (Macaca fascicularis)
Sample Types: Whole Tissue
Applications: IHC
The embryonic ontogeny of the gonadal somatic cells in mice and monkeys
Authors: K Sasaki, A Oguchi, K Cheng, Y Murakawa, I Okamoto, H Ohta, Y Yabuta, C Iwatani, H Tsuchiya, T Yamamoto, Y Seita, M Saitou
Cell Reports, 2021;35(5):109075.
Species: Cynomolgus Monkey
Sample Types: Whole Tissue
Applications: IHC"
MAdCAM1 (MECA367) in house N/A IF, ELISA Validated and published Citations: Western Blotting Analysis: A representative lot detected MAdCAM-1 in Western Blotting applications (Streeter, P.R., et. al. (1988). Nature. 331(6151):41-6); Immunohistochemistry (Paraffin) Analysis: A representative lot detected MAdCAM-1 in Immunohistochemistry applications (Streeter, P.R., et. al. (1988). Nature. 331(6151):41-6).

MAdCAM1 (MECA89) in house N/A FACS, IF Validated and published In house validation; also tested by santa cruz https://www.scbt.com/p/madcam-1-antibody-meca-89
PLVAP (MECA32) in house N/A ELISA Validated and published "Citations: Inducible expression of an endothelial cell antigen on murine myocardial vasculature in association with interstitial cellular infiltration.
Orosz CG
Transplantation 48.5 (1989 Nov): 874-7.
Novel mouse endothelial cell surface marker is suppressed during differentiation of the blood brain barrier.
Butcher EC
Developmental dynamics : an official publication of the American Association of Anatomists 202.4 (1995 Apr): 325-32."
MAdCAM1 R&D Systems AF-993 IHC, WB Validated by company via WB; https://www.rndsystems.com/products/mouse-madcam-1-antibody_af993 "Citation: Targeting improves MSC treatment of inflammatory bowel disease.
Authors: Ko IK, Kim BG, Awadallah A
Mol. Ther., 2010;18(7):1365-72.
Species: Mouse
Sample Types: Whole Cells
Applications: Neutralization"
Erg-488 Abcam 196374 PLA Validated by company via IHC; https://www.abcam.com/alexa-fluor-488-erg-antibody-epr3864-ab196374.html Citations: Brulois K et al. A molecular map of murine lymph node blood vascular endothelium at single cell resolution. Nat Commun 11:3798 (2020). PubMed: 32732867; Yanagida K et al. Sphingosine 1-Phosphate Receptor Signaling Establishes AP-1 Gradients to Allow for Retinal Endothelial Cell Specialization. Dev Cell 52:779-793.e7 (2020).
Erg-647 Abcam ab196149 IF Validated by company via IHC; https://www.abcam.com/alexa-fluor-647-erg-antibody-epr3864-ab196149.html Citation: Brulois K et al. A molecular map of murine lymph node blood vascular endothelium at single cell resolution. Nat Commun 11:3798 (2020). PubMed: 32732867
CD146 BD Biosciences 740095 IF Validated by company; https://www.bdbiosciences.com/en-us/products/reagents/flow-cytometry-reagents/research-reagents/single-color-antibodies-ruo/bv421-rat-anti-mouse-cd146.740095 Citation: Brulois K et al. A molecular map of murine lymph node blood vascular endothelium at single cell resolution. Nat Commun 11:3798 (2020). PubMed: 32732867
ly6c apc cy7 biolegend 128026 FACS Validated by company; https://www.biolegend.com/it-it/products/apc-cyanine7-anti-mouse-ly-6c-antibody-6758 Citation: Brulois K et al. A molecular map of murine lymph node blood vascular endothelium at single cell resolution. Nat Commun 11:3798 (2020). PubMed: 32732867
gp38 PeCy7 Ab Biolegend 127412 FACS Validated by company; https://www.biolegend.com/fr-lu/products/pe-cyanine7-anti-mouse-podoplanin-antibody-6674 Citation: Brulois K et al. A molecular map of murine lymph node blood vascular endothelium at single cell resolution. Nat Commun 11:3798 (2020). PubMed: 32732867
cd11b PerCP cy5.5 ab eBioscience 550993 FACS Validated by company via FACS; https://www.bdbiosciences.com/en-us/products/reagents/flow-cytometry-reagents/research-reagents/single-color-antibodies-ruo/percp-cy-5-5-rat-anti-cd11b.550993 Citation: Brulois K et al. A molecular map of murine lymph node blood vascular endothelium at single cell resolution. Nat Commun 11:3798 (2020). PubMed: 32732867
Cd11a/cd18 PerCP Cy5.5 Ab Biolegend 141008 FACS Validated by company via FACS; https://www.biolegend.com/nl-nl/products/percp-cyanine5-5-anti-mouse-cd11a-cd18-lfa-1-antibody-7043 Citation: Brulois K et al. A molecular map of murine lymph node blood vascular endothelium at single cell resolution. Nat Commun 11:3798 (2020). PubMed: 32732867
CD45 PerCP Cy5.5 Ab Biolegend 103132 FACS Validated by company via FACS Citation: Brulois K et al. A molecular map of murine lymph node blood vascular endothelium at single cell resolution. Nat Commun 11:3798 (2020). PubMed: 32732867
Nkx2-1 Ab Biopat Immunotechnologies PA0100 EMSA Validated by company via IHC, WB Citation: oven A, Morona R, González A, Moreno N. Expression patterns of Pax6 and Pax7 in the adult brain of a urodele amphibian, Pleurodeles waltl. J Comp Neurol. 2013;521:2088-124; Dominguez L, Morona R, González A, Moreno N. Characterization of the hypothalamus of Xenopus laevis during development. I. The alar regions. J Comp Neurol. 2013;521:725-59
nkx2-5 Ab Santa Cruz sc-376565X EMSA Validated by company via WB, IHC, FACs; https://datasheets.scbt.com/sc-376565.pdf Citations: Guazzi, S., et al. 1990. Thyroid nuclear factor 1 (TTF-1) contains a homeodomain and displays a novel DNA binding specificity. EMBO J. 9: 3631-3639; Komuro, I., et al. 1993. Csx: a murine homeobox-containing gene specifi- cally expressed in the developing heart. Proc. Natl. Acad. Sci. USA 90: 8145-8149.
PerCP/Cy5.5 anti-mouse TER-119/Erythroid Cells Antibody Biolegend 116228 FACS Validated by company via FACS; https://www.biolegend.com/nl-nl/products/percp-cyanine5-5-anti-mouse-ter-119-erythroid-cells-antibody-4292 Citation: Brulois K et al. A molecular map of murine lymph node blood vascular endothelium at single cell resolution. Nat Commun 11:3798 (2020). PubMed: 32732867
CD31 BV605 Biolegend 102427 FACS Validated by company via FACS; https://www.biolegend.com/en-gb/products/brilliant-violet-605-anti-mouse-cd31-antibody-9963 Citation: Brulois K et al. A molecular map of murine lymph node blood vascular endothelium at single cell resolution. Nat Commun 11:3798 (2020). PubMed: 32732867
SIRPa PerCP cy5.5 Ab Biolegend 144009 FACS Validated by company via FACS; https://www.biolegend.com/en-gb/products/percp-cyanine5-5-anti-mouse-cd172a-sirpalpha-antibody-9800 Citation: Brulois K et al. A molecular map of murine lymph node blood vascular endothelium at single cell resolution. Nat Commun 11:3798 (2020). PubMed: 32732867
ttf-1 (NKX2-1 AB) Santa Cruz SC-53136 EMSA
percpCy5.5 anti-mouse CD326 (EPCAM) clone G8.8 Biolegend 118219 FACS Validated by company via FACS; https://www.biolegend.com/ja-jp/products/percp-cyanine5-5-anti-mouse-cd326-ep-cam-antibody-5602 Citation: Brulois K et al. A molecular map of murine lymph node blood vascular endothelium at single cell resolution. Nat Commun 11:3798 (2020). PubMed: 32732867
Sambucus Nigra Lectin (SNA, EBL), Fluorescein Vector laboratories FL-1301-2 IF Validated by company via IF; https://vectorlabs.com/productpdf/download/file/id/1034/name/Sambucus_Nigra_Lectin_%2528SNA%252C_EBL%2529%252C_Fluorescein.pdf/ Citations: "Decoding Single Cell Morphology in Osteotropic Breast Cancer Cells for Dissecting Their Migratory, Molecular and Biophysical Heterogeneity" Lila Bemmerlein, Ilker A Deniz, ..., Denis Corbeil; Cancers 2022 Jan 25; "Oxygen-Dependent Changes in the N-Glycome of Murine Pulmonary Endothelial Cells" Akos Tiboldi, Johannes Führer, ..., Verena Tretter; Antioxidants 2021 Dec 04
SNA-Cy3 Vector laboratories CL-1303 IF Validated by company via IF; https://www.abcam.com/alexa-fluor-647-erg-antibody-epr3864-ab196149.html
Erg Abcam ab92513 IF Validated by company via IHC and IF; https://www.abcam.com/erg-antibody-epr3864-ab92513.html Citations: Jiang Y et al. Cerebral angiogenesis ameliorates pathological disorders in Nemo-deficient mice with small-vessel disease. J Cereb Blood Flow Metab 41:219-235 (2021); Velazquez JJ et al. Gene Regulatory Network Analysis and Engineering Directs Development and Vascularization of Multilineage Human Liver Organoids. Cell Syst 12:41-55.e11 (2021)
Nkx2-5 Santa Cruz sc-8697 IF Validated by company via IHC and WB Citations: Castagnaro, L. et al. 2013. Immunity. 38: 782-91; van

den Boogaard M, et al. 2012. J Clin Invest. 122: 2519-30
Prolong Gold antifade reagent Invitrogen P36934 IF Used for mounting for IF https://www.fishersci.com/shop/products/molecular-probes-prolong-gold-antifade-mountant-5/P36934

Eukaryotic cell lines

Policy information about cell lines and Sex and Gender in Research

Cell line source(s)	bEnd.3, HEK293T and Phoenix-Eco packaging cells were obtained from ATCC (CRL-2299, CRL-3216 and CRL-3214 respectively).
Authentication	bEnd.3 (CRL-2299): authenticated by ATCC. Citations: "Direct current stimulation modulates gene expression in isolated astrocytes with implications for glia-mediated plasticity" Limary M Cancel, Dharia Silas, ..., John M Tarbell, Scientific Reports 2022 Oct 26; "Brain Microvascular Endothelial Cell-Derived Exosomes Protect Neurons from Ischemia–Reperfusion Injury in Mice" Jin Sun, Qing Yuan, ..., Limin Hu, Pharmaceuticals 2022 Oct 19; "ROS attenuates TET2-dependent ZO-1 epigenetic expression in cerebral vascular endothelial cells" Lan Wang, Bei Mao, ..., Ying Liu, Fluids and Barriers of the CNS 2022 Sep 08<br>HEK293T (CRL-3216): authenticated by ATCC. Citations: "Hsp47 Promotes Biogenesis of Multi-subunit Neuroreceptors in the Endoplasmic Reticulum" Wang Ya-Juan, Di Xiao-Jing, bioRxiv 2022 Oct 26; "A pseudovirus-based platform to measure neutralizing antibodies in Mexico using SARS-CoV-2 as proof-of-concept" José Antonio Cruz-Cardenas, Michelle Gutierrez, ..., Marion E G Brunck, Scientific Reports 2022 Oct 26; "HSPB1 facilitates chemoresistance through inhibiting ferroptotic cancer cell death and regulating NF-κB signaling pathway in breast cancer" Liang Yiran, Wang Yajie, bioRxiv 2022 Oct 26<br>Phoenix-Eco packaging cell line (CRL-3214): authenticated by ATCC. Citations: "Antiviral effect of thiazolides relies on mitochondrial mild uncoupling" Hammad Noureddine, Ransy Céline, bioRxiv 2022 Sep 16; "Intra-peritoneal transplantation for efficient and easy generation of experimental acute myeloid leukemia in mice" Qian Fenghua, Arner Brooke E., bioRxiv 2022 Aug 05; "Quantitative Acetylomics Uncover Acetylation-Mediated Pathway Changes Following Histone Deacetylase Inhibition in Anaplastic Large Cell Lymphoma" Maša Zrimšek, Hana Kuchaříková, ..., Gerda Egger, Cells 2022 Aug 02
Mycoplasma contamination	Cell lines were tested negative for mycoplasma contamination by ATCC.
Commonly misidentified lines<br>(See ICLAC register)	No commonly misidentified cell lines were used in the study.

Animals and other research organisms

Policy information about studies involving animals; ARRIVE guidelines recommended for reporting animal research, and Sex and Gender in Research

Laboratory animals	COUP-TFIIOE/OE or COUP-TFIIKO/KO mice were crossed with CDH5(PAC)CreERT2 mice to generate tamoxifen inducible endothelial cell specific COUP-TFII OE or KO mice (EC iCOUPOE or KO). Littermates not harboring the cre transgene were used as controls. St6gal1-/- mice were from the Marth lab. Nkx2-3-/- mice were backcrossed to BALB/cJ mice and maintained at the Department of Immunology and Biotechnology, University of Pécs. C57BL/6J mice were obtained from The Jackson Laboratory, Bar Harbor, USA. All animals were housed under standard conditions, maintained in a 12 h/12 h light/dark cycle at 22 ± 2 °C and given food and tap water ad libitum. Unless otherwise stated, male and female mice between the ages of 6 to 10 weeks were used in all experiments.
Wild animals	No wild animals were used in the study.
Reporting on sex	For scRNAseq data, male and and female cohorts were processed together and resolved post-sequencing. Sex-specific data points are reported separately in relevant figures.
Field-collected samples	No field collected samples were used in the study.
Ethics oversight	All experiments were approved by the accredited Department of Laboratory Animal Medicine and the Administrative Panel on Laboratory Animal Care at Stanford and the VA Palo Alto Health Care System.

Note that full information on the approval of the study protocol must also be provided in the manuscript.
